# Potentiated Hsp104 variants suppress toxicity of diverse neurodegenerative disease-linked proteins

**DOI:** 10.1242/dmm.016113

**Published:** 2014-07-25

**Authors:** Meredith E. Jackrel, James Shorter

**Affiliations:** Department of Biochemistry and Biophysics, Perelman School of Medicine at the University of Pennsylvania, Philadelphia, PA 19104, USA.

**Keywords:** FUS, Hsp104, TDP-43, α-synuclein, Disaggregase, Neurodegeneration

## Abstract

Protein misfolding is implicated in numerous lethal neurodegenerative disorders, including amyotrophic lateral sclerosis (ALS) and Parkinson disease (PD). There are no therapies that reverse these protein-misfolding events. We aim to apply Hsp104, a hexameric AAA+ protein from yeast, to target misfolded conformers for reactivation. Hsp104 solubilizes disordered aggregates and amyloid, but has limited activity against human neurodegenerative disease proteins. Thus, we have previously engineered potentiated Hsp104 variants that suppress aggregation, proteotoxicity and restore proper protein localization of ALS and PD proteins in *Saccharomyces cerevisiae*, and mitigate neurodegeneration in an animal PD model. Here, we establish that potentiated Hsp104 variants possess broad substrate specificity and, in yeast, suppress toxicity and aggregation induced by wild-type TDP-43, FUS and α-synuclein, as well as missense mutant versions of these proteins that cause neurodegenerative disease. Potentiated Hsp104 variants also rescue toxicity and aggregation of TAF15 but not EWSR1, two RNA-binding proteins with a prion-like domain that are connected with the development of ALS and frontotemporal dementia. Thus, potentiated Hsp104 variants are not entirely non-specific. Indeed, they do not unfold just any natively folded protein. Rather, potentiated Hsp104 variants are finely tuned to unfold proteins bearing short unstructured tracts that are not recognized by wild-type Hsp104. Our studies establish the broad utility of potentiated Hsp104 variants.

## INTRODUCTION

Fatal neurodegenerative diseases including amyotrophic lateral sclerosis (ALS), Parkinson disease (PD), Huntington disease (HD) and Alzheimer disease (AD) are classified as protein-misfolding disorders (Cushman et al., 2010; Dobson, 2003). Each of these disorders is associated with the accumulation of misfolded aggregates and amyloids comprised of different proteins. In PD, α-synuclein (α-syn) accumulates in highly toxic pre-fibrillar oligomers and amyloid fibers (Cushman et al., 2010). In ALS, several different RNA-binding proteins with prion-like domains, including TDP-43, FUS, EWSR1 and TAF15, are implicated in the disease in certain subsets of affected individuals (Couthouis et al., 2012; Couthouis et al., 2011; King et al., 2012; Robberecht and Philips, 2013). Although normally nuclear proteins, in the disease state, TDP-43, FUS, EWSR1 and TAF15 accumulate in cytoplasmic inclusions in the degenerating motor neurons of individuals with ALS (Couthouis et al., 2012; Couthouis et al., 2011; Robberecht and Philips, 2013). Additionally, in ~15% of ALS-affected individuals, this neurodegeneration is accompanied by frontotemporal dementia (FTD) (Robberecht and Philips, 2013). These aggregates are widely considered intractable, and no available therapy eliminates these structures or their misfolded precursors. Accordingly, treatments for these disorders remain palliative. Therapeutics that eliminate these misfolded conformers by degrading them or reactivating them to their native fold are urgently needed.

Not all protein-misfolding events, including amyloidogenesis, are associated with toxicity. In *Saccharomyces cerevisiae*, amyloid-based prions are tightly regulated and utilized for various adaptive purposes (Garcia and Jarosz, 2014; Newby and Lindquist, 2013). Hsp104, a hexameric AAA+ protein from yeast, catalyzes construction and deconstruction of amyloid and pre-fibrillar conformers (Shorter and Lindquist, 2004; Shorter and Lindquist, 2006; Shorter and Lindquist, 2008). Hsp104 also reactivates proteins that are trapped in disordered aggregates after environmental stress (Glover and Lindquist, 1998; Parsell et al., 1994; Parsell et al., 1991; Sanchez and Lindquist, 1990; Shorter, 2008). Although highly conserved in eubacteria and eukaryotes, Metazoa lack an Hsp104 homolog and have limited capabilities for disaggregating disordered and amyloid aggregates (Duennwald et al., 2012; Shorter, 2008; Shorter, 2011; Torrente and Shorter, 2013). Thus, we hypothesized that yeast Hsp104 could be harnessed to counter the protein misfolding that is implicated in human neurodegenerative disease (Shorter, 2008; Vashist et al., 2010). However, Hsp104 has only limited ability to disaggregate human neurodegenerative disease proteins, which it does not ordinarily encounter (DeSantis et al., 2012; Lo Bianco et al., 2008). Thus, we have previously engineered potentiated Hsp104 variants to eradicate TDP-43, FUS and α-syn aggregates (Jackrel et al., 2014; Jackrel and Shorter, 2014). These variants potently suppress toxicity in situations where wild-type Hsp104 (Hsp104^WT^) is ineffective (Jackrel et al., 2014). Additionally, TDP-43 and α-syn are not only refolded by potentiated Hsp104, but are also returned to their proper cellular localization (Jackrel et al., 2014). Furthermore, we have previously demonstrated that two of the potentiated variants, Hsp104^A503S^ and Hsp104^DPLF-A503V^, prevent dopaminergic neurodegeneration in a *Caenorhabditis elegans* model of PD (Jackrel et al., 2014).

Numerous missense mutations in TDP-43, FUS and α-syn have been identified and implicated in ALS and PD, respectively (Pesiridis et al., 2009; Polymeropoulos et al., 1997; Robberecht and Philips, 2013; Zarranz et al., 2004). Many of these variants have been linked to aggressive, early-onset forms of disease, enhanced proteotoxicity and an increased propensity to aggregate *in vitro* and in animal models (Choi et al., 2004; Conway et al., 2000; Fredenburg et al., 2007; Johnson et al., 2009; Qiu et al., 2014; Sun et al., 2011). Therefore, it is crucial to test whether the potentiated Hsp104 variants rescue the toxicity and aggregation of these disease-linked variants. Here, we demonstrate that the potentiated Hsp104 variants are active against a broad range of disease-linked TDP-43, FUS and α-syn mutants. Moreover, potentiated Hsp104 variants, but not Hsp104^WT^, rescue TAF15 toxicity and aggregation. However, these Hsp104 variants are not entirely non-specific, as they do not suppress the toxicity or aggregation associated with EWSR1 (Couthouis et al., 2012; Couthouis et al., 2011; Robberecht and Philips, 2013). To better understand the basis of Hsp104 potentiation, we have also used pure protein biochemistry to probe the minimum requirements for substrate recognition by Hsp104 and its potentiated variants. Our studies provide new insights into the basis for potentiated Hsp104 activity against a diverse array of substrates and provide clues as to how potentiated Hsp104 might be fine-tuned to enhance its substrate specificity.

**TRANSLATIONAL IMPACT**

**Clinical issue**

Protein misfolding is implicated in numerous lethal neurodegenerative disorders, including amyotrophic lateral sclerosis (ALS), Parkinson disease (PD) and frontotemporal dementia (FTD). Each of these disorders is associated with the accumulation of misfolded aggregates and amyloids comprised of different proteins. For example, several RNA-binding proteins with prion-like domains, including TDP-43, FUS, TAF15 and EWSR1, have been implicated in ALS. These normally nuclear proteins accumulate in cytoplasmic inclusions in degenerating motor neurons in individuals with ALS. Similarly, in PD, α-synuclein accumulates in highly toxic pre-fibrillar oligomers and amyloid fibers. There are no therapies that reverse these protein-misfolding events, and treatments for these neurodegenerative disorders remain palliative.

**Results**

Hsp104 is a hexameric AAA+ protein from yeast that targets misfolded conformers for reactivation. Although Hsp104 solubilizes disordered aggregates and amyloid, it has limited activity against human neurodegenerative disease proteins. In this study, the authors investigate the substrate specificity of a previously engineered set of potentiated Hsp104 variants that suppress aggregation and proteotoxicity, restore the proper localization of some proteins implicated in ALS, FTD and PD in yeast, and mitigate neurodegeneration in an animal model of PD. They report that, in yeast, potentiated Hsp104 variants potently suppressed toxicity induced by wild-type TDP-43, FUS and α-synuclein, as well as toxicity induced by missense mutant versions of these proteins that cause neurodegenerative disease. Potentiated Hsp104 variants also rescued toxicity and aggregation of TAF15 but not EWSR1.

**Implications and future directions**

This study establishes the broad utility of potentiated Hsp104 variants, by demonstrating the ability of these enhanced disaggregases to eliminate diverse amyloid and non-amyloid disease-associated aggregates. More generally, this study demonstrates that large proteins (like Hsp104), whose structures remain poorly understood, are viable candidates for protein engineering. Notably, potentiated Hsp104 variants are not entirely non-specific but are finely tuned to unfold proteins bearing short unstructured tracts that are not recognized by wild-type Hsp104. Finally, by demonstrating that neurodegenerative disease phenotypes can be suppressed and even reversed, the results presented in this study represent a key advance in the development of new potential strategies to treat protein-misfolding disorders.

## RESULTS

### Potentiated Hsp104 variants suppress toxicity of ALS-linked TDP-43 mutants

ALS is primarily a sporadic disorder, but numerous missense mutations in TDP-43 have been identified and linked to ALS (Gitcho et al., 2008; Pesiridis et al., 2009; Sreedharan et al., 2008). We have identified several potentiated Hsp104 variants that suppress the toxicity of wild-type TDP-43 (TDP-43^WT^) (Jackrel et al., 2014). Thus, we were curious as to whether they could also suppress the toxicity of the ALS-linked TDP-43 variants TDP-43^A315T^, TDP-43^Q331K^ and TDP-43^M337V^ (Gitcho et al., 2008; Sreedharan et al., 2008). Each of these TDP-43 mutations (denoted by the superscripts) is located in the C-terminal prion-like domain of TDP-43 (Cushman et al., 2010; Johnson et al., 2009). TDP-43^A315T^ and TDP-43^M337V^ have been linked to familial ALS, whereas TDP-43^Q331K^ has been linked to sporadic ALS (Gitcho et al., 2008; Sreedharan et al., 2008). TDP-43^Q331K^ and TDP-43^M337V^ aggregate more rapidly than TDP-43^WT^ *in vitro* and are more toxic and form more aggregates per cell when overexpressed in yeast as compared with TDP-43^WT^ (Johnson et al., 2009). TDP-43^A315T^ is also more aggregation-prone than TDP-43^WT^ *in vitro* and *in vivo* (Guo et al., 2011).

**Fig. 1.**
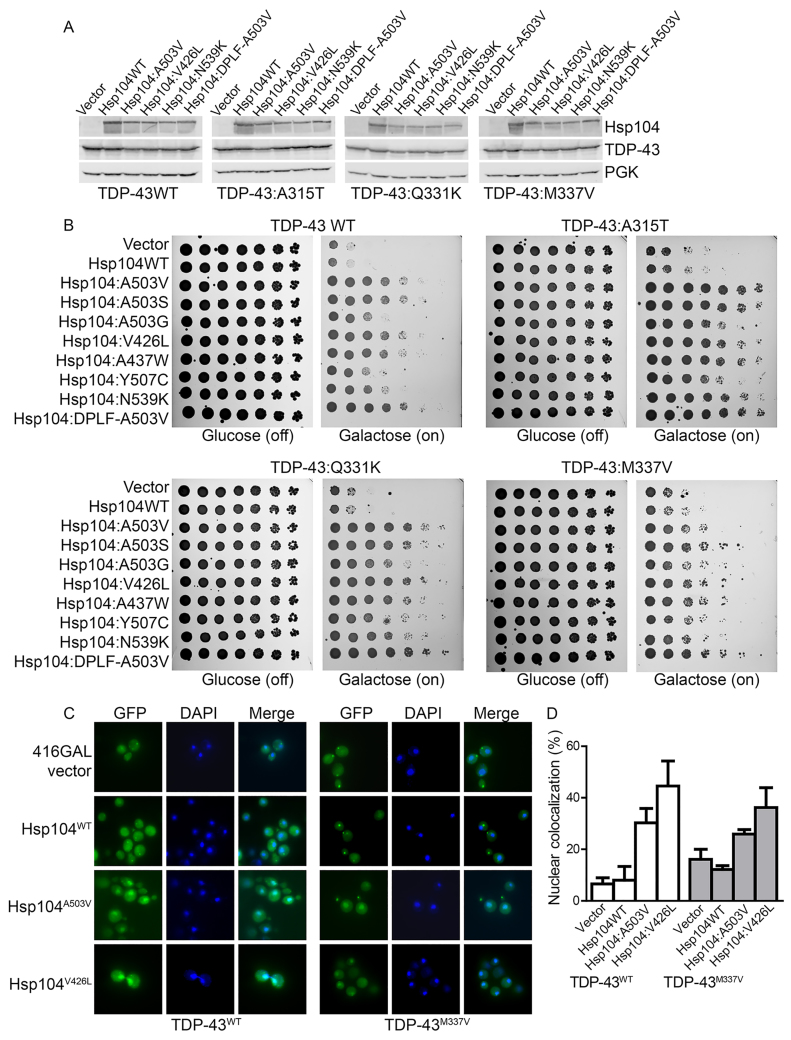
**Potentiated Hsp104 variants rescue toxicity of ALS-linked TDP-43 mutants.** (A) Selected strains from B expressing the indicated constructs were induced for 5 h, lysed and then immunoblotted for the specified proteins. A loading control, 3-phosphoglycerate kinase (PGK), was used. (B) Δ*hsp104* yeast integrated with the indicated pAG303GAL-TDP-43 variant were transformed with the indicated pAG416GAL-Hsp104 variant or vector control. Strains were serially diluted fivefold and spotted on glucose (off) or galactose (on) medium. (C) Fluorescence microscopy of cells co-expressing fluorescently tagged TDP-43 constructs (TDP-43^WT^, left; TDP-43^M337V^, right) and Hsp104^WT^, Hsp104^A503V^ Hsp104^V426L^ or vector (416Gal). Cells were stained with DAPI to visualize nuclei (blue). (D) TDP-43 localization was quantified by counting the number of cells containing colocalized nuclear staining. Values represent means±s.e.m. (*n*=3).

Plasmids harboring these TDP-43 mutants have been previously integrated into Δ*hsp104 Saccharomyces cerevisiae*, and then the potentiated Hsp104 variants Hsp104^A503V^, Hsp104^A503S^, Hsp104^A503G^, Hsp104^V426L^, Hsp104^A437W^, Hsp104^Y507C^, Hsp104^N539K^ and Hsp104^DPLF-A503V^ (Jackrel et al., 2014) were transformed into these strains. These Hsp104 variants were selected to encompass a range of missense mutations in helices 1, 2 and 3 of the middle domain, as well as the small domain of NBD1 (Jackrel et al., 2014). We utilized a Δ*hsp104* yeast strain to avoid expression of endogenous Hsp104^WT^. However, in this background, the differences in toxicity between TDP-43^WT^ and TDP-43^Q331K^ are not as pronounced as in the wild-type yeast background (Johnson et al., 2009). Additionally, in this background, TDP-43^M337V^ is not as toxic as in the wild-type strain, indicating that Hsp104^WT^ can modulate TDP-43 toxicity (Kim et al., 2014). We confirmed that the Hsp104 variants do not alter the expression levels of TDP-43 or its variants and that they are expressed at similar or lower levels than Hsp104^WT^ (Fig. 1A). The potentiated Hsp104 variants potently suppressed the toxicity of TDP-43^A315T^ and TDP-43^Q331K^ (Fig. 1B). Hsp104 variants also suppressed the toxicity of TDP-43^M337V^, although this rescue was not as strong as for the other TDP-43 variants (Fig. 1B). Hsp104^WT^ did not modify the toxicity of any of the TDP-43 variants (Fig. 1B). The toxicity suppression conferred by Hsp104^A503G^ and Hsp104^Y507C^ was less pronounced, whereas Hsp104^A503V^, Hsp104^A503S^, Hsp104^V426L^, Hsp104^A437W^, Hsp104^N539K^ and Hsp104^DPLF-A503V^ suppressed toxicity more strongly (Fig. 1B). Thus, potentiated Hsp104 variants suppressed the toxicity of TDP-43^WT^, which forms cytoplasmic aggregates in the vast majority (~95%) of ALS cases, as well as the toxicity of rare ALS-linked TDP-43 variants that are found in sporadic and familial forms of ALS (Lagier-Tourenne et al., 2010; Robberecht and Philips, 2013; Sreedharan et al., 2008).

TDP-43 normally shuttles between the nucleus and cytoplasm; however, in ALS, TDP-43 is typically depleted from the nucleus and aggregates in the cytoplasm (Robberecht and Philips, 2013). Using fluorescence microscopy, we have previously demonstrated that Hsp104^A503V^ both eliminates cytoplasmic TDP-43 aggregates and promotes nuclear TDP-43 localization (Jackrel et al., 2014). Thus, we tested whether the potentiated Hsp104 variants have similar effects in yeast expressing the ALS-linked TDP-43^M337V^ variant. We selected Hsp104^A503V^ and Hsp104^V426L^ for further analysis using fluorescence microscopy, as both of these variants strongly suppressed the toxicity of both TDP-43^WT^ and the ALS-linked variants. Here, as expected, cytoplasmic aggregates of both TDP-43^WT^ and TDP-43^M337V^ persisted upon Hsp104^WT^ overexpression (Fig. 1C). Hsp104^A503V^ and Hsp104^V426L^ eliminated some of the cytoplasmic TDP-43^WT^ aggregates and ~30% and ~45% of cells, respectively, had nuclear TDP-43 localization, compared with ~6–8% in the vector and Hsp104^WT^ controls (Fig. 1D). Similarly, for TDP-43^M337V^, ~26% and ~36% of cells, respectively, had nuclear TDP-43 localization (Fig. 1D). Thus, Hsp104^A503V^ and Hsp104^V426L^ can rescue the toxicity and nuclear localization of TDP-43 and ALS-linked variants.

### Potentiated Hsp104 variants suppress the toxicity of ALS-linked FUS mutants

**Fig. 2.**
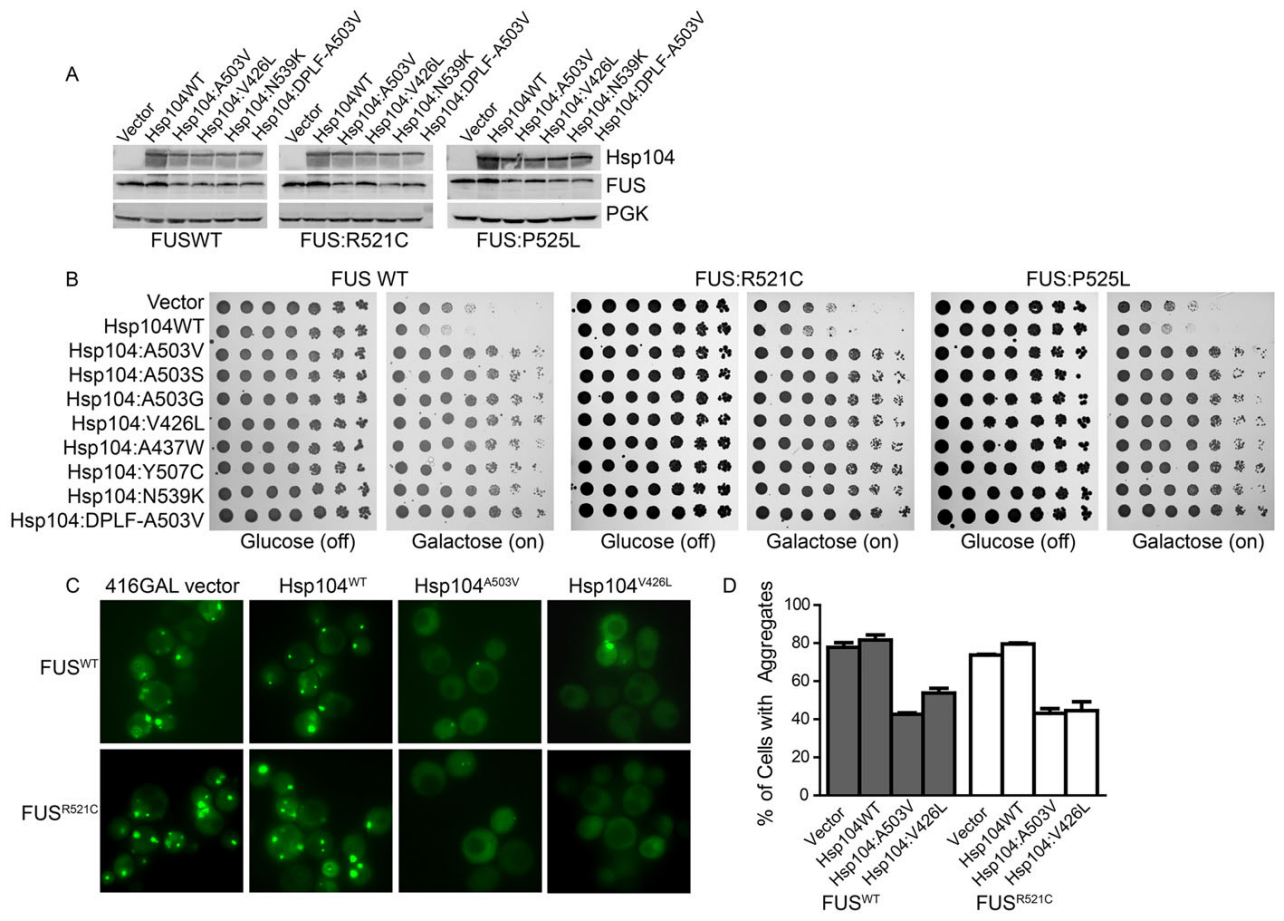
**Potentiated Hsp104 variants rescue toxicity of ALS-linked FUS mutants.** (A) Selected strains from B expressing the indicated constructs were induced for 5 h, lysed and then immunoblotted for the specified proteins. PGK serves as a loading control. (B) Δ*hsp104* yeast were co-transformed with the indicated pAG413GAL-FUS variant and the indicated pAG416GAL-Hsp104 variant or vector control. Strains were serially diluted fivefold and spotted on glucose (off) or galactose (on) medium. (C) Fluorescence microscopy of cells co-expressing FUS-GFP or FUS^R521C^-GFP and Hsp104^WT^, Hsp104^A503V^, Hsp104^V426L^ or vector (416GAL). (D) FUS aggregation was quantified by counting the number of cells containing 0 or 1 or more foci. Values represent means±s.e.m. (*n*=2–3).

Next, we tested whether potentiated Hsp104 variants could also suppress the toxicity of the ALS-linked FUS variants. We tested FUS^P525L^ and FUS^R521C^, where both proteins harbor mutations in the proline-tyrosine nuclear localization signal (NLS) of FUS (Dormann et al., 2010; Huang et al., 2010; Qiu et al., 2014; Robberecht and Philips, 2013; Vance et al., 2009). FUS^R521C^ aggregates with similar kinetics to FUS^WT^ *in vitro*, and FUS^P525L^ and FUS^R521C^ exhibit similar toxicity and aggregation properties to FUS^WT^ in yeast (Sun et al., 2011). Plasmids harboring these FUS mutants and the series of Hsp104 variants were co-transformed in Δ*hsp104* yeast. We first tested whether the variants alter expression levels of FUS. We found that the Hsp104 variants slightly reduced FUS expression levels (Fig. 2A). However, the Hsp104 variants were expressed at lower levels than Hsp104^WT^ (Fig. 2A). The potentiated Hsp104 variants potently suppressed toxicity of both FUS mutants rather uniformly, whereas Hsp104^WT^ and vector alone did not modify the toxicity of FUS or either ALS-linked FUS mutant (Fig. 2B). Thus, potentiated Hsp104 variants suppress the toxicity of FUS^WT^, which forms cytoplasmic aggregates in FTD-affected individuals, as well as the toxicity of rare ALS-linked FUS variants that are found in familial forms of ALS (Lagier-Tourenne et al., 2010; Robberecht and Philips, 2013).

We then tested whether the potentiated Hsp104 variants suppressed the aggregation of a disease-linked FUS variant. We utilized FUS-GFP (green fluorescent protein) and fluorescence microscopy (Fig. 2C,D) to assess the formation of FUS aggregates. We selected Hsp104^A503V^ and Hsp104^V426L^ for further analyses, as each of these variants strongly suppressed the toxicity of both of the ALS-linked FUS mutants (Fig. 2B). Here, as expected (Jackrel et al., 2014; Sun et al., 2011), FUS^WT^ aggregated strongly, with ~80% of cells displaying cytoplasmic aggregates (Fig. 2C,D). Hsp104^A503V^ and Hsp104^V426L^ prevented aggregate accumulation, with only ~43% and ~54% of cells containing aggregates, respectively (Fig. 2C,D). Similar results were obtained with FUS^R521C^, which also aggregated in ~80% of the cells harboring the vector or the Hsp104^WT^ control (Fig. 2C,D). Hsp104^A503V^ and Hsp104^V426L^ suppressed FUS^R521C^ aggregation with ~43% and ~45% of cells containing aggregates, respectively (Fig. 2C,D). These results correspond well with the toxicity assays in which FUS^WT^ and FUS^R521C^ exhibited similar levels of toxicity, and both are rescued strongly by Hsp104^A503V^ and Hsp104^V426L^.

### Potentiated Hsp104 variants suppress the toxicity and aggregation of PD-linked α-synuclein mutants

**Fig. 3.**
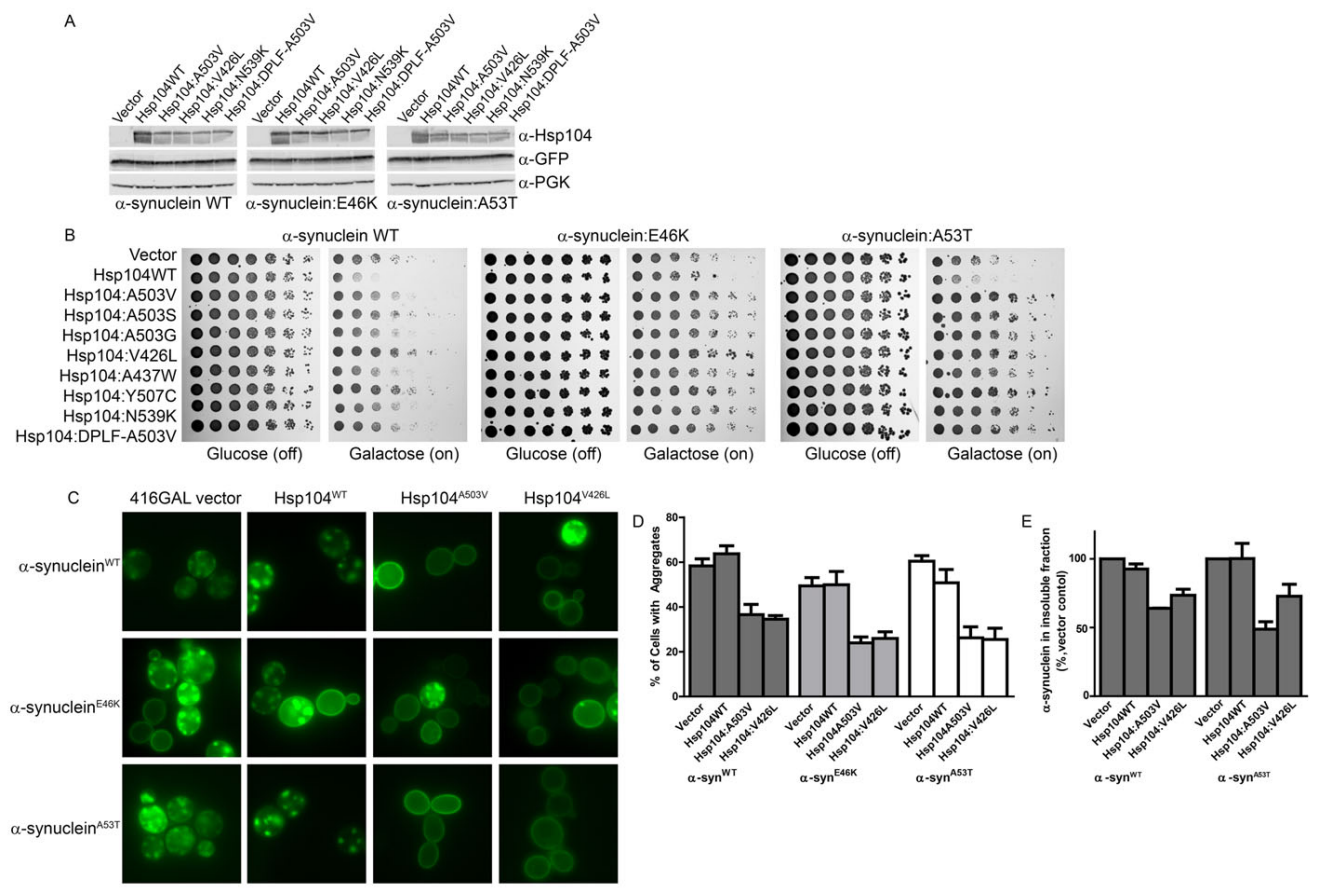
**Potentiated Hsp104 variants rescue PD-linked α-synuclein mutants.** (A) Selected strains from B expressing the indicated constructs were induced for 8 h, lysed and then immunoblotted for the specified proteins. α-syn-YFP is detected using an antibody against GFP. PGK serves as a loading control. (B) Δ*hsp104* yeast were co-transformed with the indicated pAG423GAL-α-syn-YFP variant and the indicated pAG416GAL-Hsp104 variant or vector control. Strains were serially diluted fivefold and spotted on glucose (off) or galactose (on) medium. (C) Selected strains from B were induced and analyzed by fluorescence microscopy for aggregates of the indicated α-syn variant (green). (D) Quantification of the percentage of cells from C that displayed cytoplasmic aggregates. Values represent means±s.e.m. (*n*= 3–4). (E) Selected strains from B were induced for 8h, lysed and then processed for sedimentation analysis and quantitative immunoblot. The relative amount of insoluble to total α-syn was determined as a percentage of that of the vector control. Values represent means±s.e.m. (*n*=2).

PD is primarily a sporadic disorder, but mutations in α-syn (including α-syn^E46K^ and α-syn^A53T^), as well as higher expression levels of α-syn^WT^, have been linked to PD (Bendor et al., 2013; Jones et al., 2014; Recchia et al., 2004). α-Syn^E46K^ and α-syn^A53T^ fibrillize faster than α-syn^WT^, and although α-syn^A53T^ accesses pre-amyloid oligomeric species more rapidly than α-syn^WT^, α-syn^E46K^ is less able to access these species (Choi et al., 2004; Conway et al., 2000; Fredenburg et al., 2007). Plasmids harboring these α-syn variants and Hsp104 were co-transformed into Δ*hsp104* yeast. We utilized 2μ plasmids for expression of α-syn in these experiments, and these constructs display greater toxicity than the doubly integrated strain that we used for our initial screens to identify the potentiated variants (Jackrel et al., 2014; Outeiro and Lindquist, 2003). Thus, α-syn^WT^ is more toxic in this setting than in our previous work (Jackrel et al., 2014). We confirmed that the Hsp104 variants do not alter expression of the α-syn variants and that the Hsp104 variants are expressed at similar or lower levels than Hsp104^WT^ (Fig. 3A). Each of the Hsp104 variants tested slightly suppressed the toxicity of α-syn^WT^, and Hsp104^A503S^, Hsp104^V426L^ and Hsp104^Y507C^ conferred the strongest rescue (Fig. 3B). By contrast, the potentiated Hsp104 variants strongly suppressed the toxicity of α-syn^E46K^ (Fig. 3B) and even more potently suppressed the toxicity of α-syn^A53T^ (Fig. 3B). Hsp104^WT^ and vector did not strongly modify the toxicity of either α-syn variant, although Hsp104^WT^ slightly enhanced the toxicity of α-syn^WT^ and α-syn^A53T^ (Fig. 3B).

Next, we tested whether the potentiated Hsp104 variants suppress the aggregation of the α-syn variants. We utilized yellow fluorescent protein (YFP)-tagged α-syn and fluorescence microscopy (Fig. 3C,D) to assess the formation of α-syn aggregates. We selected Hsp104^A503V^ and Hsp104^V426L^ for microscopy, as these variants strongly suppressed the toxicity of both PD-linked α-syn mutants (Fig. 3B). Here, as expected (Jackrel et al., 2014; Outeiro and Lindquist, 2003), α-syn^WT^ aggregated strongly, with ~60% of cells displaying cytoplasmic aggregates (Fig. 3C,D). Hsp104^A503V^ and Hsp104^V426L^ prevented aggregate accumulation, with only ~37% and ~35% of cells containing aggregates (Fig. 3C,D). Moreover, Hsp104^A503V^ and Hsp104^V426L^ increased α-syn^WT^ localization to the plasma membrane (Fig. 3C). This rescue was surprising, as these variants only slightly suppressed the toxicity of α-syn^WT^ in this strain (Fig. 3B). α-Syn^E46K^ aggregated in ~50% of cells harboring the vector or Hsp104^WT^ control (Fig. 3C,D). Hsp104^A503V^ and Hsp104^V426L^ suppressed α-syn^E46K^ aggregation with ~24% and ~26% of cells containing aggregates, respectively (Fig. 3C,D). Hsp104^A503V^ and Hsp104^V426L^ also increased α-syn^E46K^ localization to the plasma membrane (Fig. 3C). α-syn^A53T^ aggregated in a similar proportion of cells to that of α-syn^WT^ when co-expressed with the vector or Hsp104^WT^, with ~60% and ~51% of cells displaying cytoplasmic aggregates, respectively (Fig. 3C,D). Both Hsp104^A503V^ and Hsp104^V426L^ suppressed aggregation, with only ~26% of cells containing aggregates when co-expressing either variant (Fig. 3D). Hsp104^A503V^ and Hsp104^V426L^ also increased α-syn^A53T^ localization to the plasma membrane (Fig. 3C). Accordingly, sedimentation analysis revealed that Hsp104^A503V^ and Hsp104^V426L^ reduced the amount of insoluble α-syn^WT^ by ~36% and ~27%, respectively. By contrast, Hsp104^WT^ reduced the amount of insoluble α-syn^WT^ by ~7% (Fig. 3E). Hsp104^A503V^ and Hsp104V^426L^ also reduced the amount of insoluble α-syn^A53T^ by ~51% and ~27%, respectively, whereas Hsp104^WT^ did not alter α-syn^A53T^ solubility (Fig. 3E). Thus, potentiated Hsp104 variants suppress aggregation, increase solubility and enable plasma membrane localization of PD-linked α-syn^A53T^ and α-syn^E46K^ in yeast.

### Potentiated Hsp104 variants do not suppress EWSR1 toxicity or aggregation

**Fig. 4.**
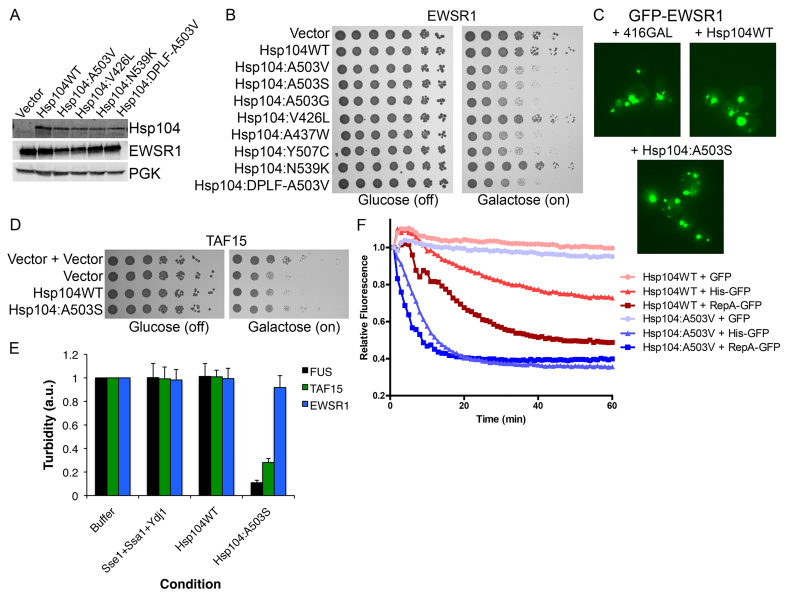
**Potentiated Hsp104 variants rescue TAF15 but not EWSR1 aggregation and toxicity.** (A) Selected strains from B were induced for 5 h, lysed and immunoblotted for the specified proteins. (B) Δ*hsp104* yeast were co-transformed with pAG423GAL-EWSR1 and the indicated pAG416GAL-Hsp104 variant or vector control. Strains were serially diluted fivefold and spotted on glucose (off) or galactose (on) medium. (C) Δ*hsp104* yeast were co-transformed with pAG426GAL-GFP-EWSR1 and the indicated pAG413GAL-Hsp104 variant or vector control. Yeast were induced in galactose for 5 h and then harvested for fluorescence microscopy analyses of EWSR1 (green) aggregation. (D) Δ*hsp104* yeast were co-transformed with pAG426GAL-TAF15 and the indicated pAG413GAL-Hsp104 variant or vector control. Strains were serially diluted tenfold and spotted on glucose (off) or galactose (on) medium. (E) Preformed FUS (black bars), TAF15 (green bars) or EWSR1 (blue bars) aggregates were incubated with Hsp104^WT^ or Hsp104^A503S^ with Ssa1, Ydj1 and Sse1 for 60 min at 30°C. Controls with buffer or Sse1, Ssa1 and Ydj1 alone were also included. Aggregate dissolution was assessed by turbidity (a.u., arbitrary units). Values represent means±s.e.m. (*n*=3). (F) Hsp104^A503V^ unfolds substrates with shorter unfolded tracts, compared with Hsp104^WT^. RepA_1–70_-GFP, 6-HIS-GFP or GFP was incubated with Hsp104^WT^ or Hsp104^A503V^ and GroEL^trap^ plus ATP (for Hsp104^A503V^) or ATP:ATPγS (3:1, for Hsp104^WT^). GFP unfolding was assessed via fluorescence. Representative data sets are shown.

We next investigated whether these Hsp104 variants could suppress toxicity in other yeast proteotoxicity models. Thus, we tested them against EWSR1, another RNA-binding protein with a prion-like domain that is implicated in ALS and FTD (Couthouis et al., 2012; Couthouis et al., 2011; King et al., 2012; Mackenzie and Neumann, 2012; Robberecht and Philips, 2013). When overexpressed in yeast, EWSR1 is toxic and forms cytoplasmic aggregates, similar to FUS and TDP-43 (Couthouis et al., 2012; Couthouis et al., 2011). Plasmids harboring the Hsp104 variants were co-transformed with an EWSR1 plasmid into yeast. We utilized 2μ plasmids to give high levels of EWSR1 expression. We tested the effects of Hsp104 expression on EWSR1 expression and found that EWSR1 levels remained constant, except for somewhat decreased levels when co-expressed with Hsp104^A503V^ and Hsp104^V426L^ (Fig. 4A). However, each of the Hsp104 variants were expressed at lower levels than Hsp104^WT^ (Fig. 4A). Surprisingly, none of the potentiated variants suppressed EWSR1 toxicity and instead enhanced toxicity, with the exception of Hsp104^V426L^ and Hsp104^N539K^, which did not modify toxicity (Fig. 4B). To confirm that the variants were inactive against EWSR1, we assessed GFP-EWSR1 aggregation in yeast (Fig. 4C). GFP-EWSR1 aggregated strongly in yeast, and expression of the vector, Hsp104^WT^ or Hsp104^A503S^ did not suppress this aggregation (Fig. 4C). Thus, the potentiated Hsp104 variants are not able to rescue the toxicity or aggregation of all human neurodegenerative disease proteins in yeast. Nonetheless, our experimental paradigm now provides a platform to isolate potentiated Hsp104 variants that rescue EWSR1 aggregation and toxicity.

### Potentiated Hsp104 variants suppress TAF15 toxicity and aggregation

It is intriguing that potentiated Hsp104 variants suppress FUS toxicity but can enhance EWSR1 toxicity. Thus, we tested potentiated Hsp104 variants against TAF15, which is closely related to FUS and EWSR1 and is also connected with ALS and FTD (Couthouis et al., 2012; Couthouis et al., 2011; King et al., 2012). TAF15 was not as toxic in yeast as FUS or EWSR1 (Fig. 4D) (Couthouis et al., 2012), but Hsp104^A503S^ suppressed this toxicity, returning growth to levels similar to those of yeast harboring the vector alone (Fig. 4D). To assess this selective activity against FUS and TAF15 further, we tested whether Hsp104^A503S^ could disaggregate preformed FUS, TAF15 and EWSR1 aggregates *in vitro* (Couthouis et al., 2012). Remarkably, Hsp104^A503S^, but not Hsp104^WT^, could disaggregate preformed FUS and TAF15 aggregates in the presence of Hsp110 (Sse1), Hsp70 (Ssa1) and Hsp40 (Ydj1), which were inactive alone (Fig. 4E). By contrast, neither Hsp104^A503S^ nor Hsp104^WT^ could disaggregate EWSR1 (Fig. 4E). Thus, EWSR1 aggregates appeared to be refractory to Hsp104^A503S^. This finding helps explain why Hsp104^A503S^ can rescue FUS and TAF15, but not EWSR1 toxicity in yeast.

### Potentiated Hsp104^A503V^ selects appropriate substrates for unfolding in a different manner to Hsp104^WT^

It is surprising that the enhanced Hsp104 variants potently disaggregated dissimilar disordered aggregates and amyloid fibrils, whereas Hsp104^WT^ was inactive. Thus, we sought to further explore the substrate recognition properties of the potentiated variants in comparison with Hsp104^WT^. To do so, we utilized a GFP-unfolding assay with purified proteins, which allows monitoring of the unfolding of a GFP substrate through loss of fluorescence over time (Doyle et al., 2007). We first compared Hsp104^WT^ and Hsp104^A503V^ in the unfolding of RepA_1–70_-GFP. Here, RepA_1-70_ serves as a 70-amino-acid unfolded tract appended to GFP (Hoskins et al., 2000). Hsp104^WT^ strongly unfolded RepA_1–70_-GFP, but only in the presence of a 3:1 ratio of ATP:ATPγS, where ATPγS promotes Hsp104 unfoldase activity in the absence of Hsp70 and Hsp40 (Doyle et al., 2007). In the presence of just ATP, Hsp104^WT^ does not unfold RepA_1–70_-GFP (Doyle et al., 2007; Jackrel et al., 2014). Hsp104^A503V^ unfolded RepA_1–70_-GFP more rapidly than Hsp104^WT^ in the presence of ATP alone and did not require ATPγS (Fig. 4F). We next tested the ability of the variants to unfold a substrate with a shorter unfolded tract, 6-HIS-GFP, in which a tract of six histidine residues are appended to GFP through a TEV protease cleavage site linker. Hsp104^WT^ did not robustly unfold this substrate in the presence of ATP:ATPγS (Fig. 4F). In stark contrast, Hsp104^A503V^ unfolded it as rapidly as it did the RepA_1–70_-GFP substrate in the presence of ATP alone (Fig. 4F). This difference indicates that Hsp104^A503V^ is a more powerful unfoldase that recognizes even very short unfolded regions of substrates with greater precision than Hsp104^WT^. Finally, we cleaved the 6-HIS tag from GFP using TEV protease and tested the proteins for the ability to unfold untagged GFP. Here, neither Hsp104^WT^ nor Hsp104^A503V^ unfolded GFP, indicating that Hsp104^A503V^ does not unfold any natively folded protein (Fig. 4F). Rather, Hsp104^A503V^ is finely tuned to distinguish between fully folded and subtly destabilized proteins, whereas Hsp104^WT^ requires a greater difference to recognize substrates for unfolding.

## DISCUSSION

Here, we demonstrate that potentiated Hsp104 variants powerfully suppress the toxicity of a diverse array of substrates implicated in ALS, FTD and PD. These variants not only suppress the toxicity of the wild-type proteins, which misfold in sporadic forms of disease, but they also suppress the toxicity conferred by the corresponding disease-associated mutants, which are connected with rarer sporadic cases and familial forms of disease. Thus, we have established that Hsp104^A503V^ and Hsp104^V426L^ suppress aggregation and restore plasma membrane localization of α-syn^A53T^ and α-syn^E46K^, which are associated with early-onset forms of familial PD (Polymeropoulos et al., 1997; Recchia et al., 2004). We have also found that Hsp104^A503V^ and Hsp104^V426L^ reduce toxicity, suppress aggregation and promote the nuclear localization of not only TDP-43^WT^ but also the ALS-linked TDP-43^M337V^ variant (Sreedharan et al., 2008). Hsp104^A503V^ and Hsp104^V426L^ also reduce the toxicity and suppress the aggregation of ALS-linked FUS^R521C^ (Vance et al., 2009). Moreover, Hsp104^A503S^ rescues the toxicity and aggregation of TAF15, which is connected to ALS and FTD (Couthouis et al., 2011; Mackenzie and Neumann, 2012). However, these Hsp104 variants are not uniformly potentiated against all of the substrates that they encounter, as they remain unable to suppress the toxicity or aggregation of EWSR1, which is also connected to ALS and FTD (King et al., 2012; Mackenzie and Neumann, 2012).

In some cases, potentiated Hsp104 variants yielded a modest reduction in the amount of insoluble protein, as well as the proportion of cells with aggregates, but they could also yield a large rescue of toxicity (Fig. 3D,E). There are several possible explanations for this observation. First, a comparatively modest reduction in global levels of protein aggregation below a certain threshold in all cells could be sufficient to rescue cell viability. Second, the potentiated Hsp104 variants could selectively resolve the most toxic aggregated conformers or ‘strains’ and leave more benign aggregated structures intact (Cushman-Nick et al., 2013; DeSantis and Shorter, 2012). Thus, the remaining aggregated structures could be much less toxic. Third, potentiated Hsp104 variants could eliminate small, soluble oligomeric species formed by disease proteins that can be highly toxic (DeSantis et al., 2012; Lo Bianco et al., 2008). Elimination of these toxic oligomeric species could yield big increases in viability, whereas any aggregated species that remain might be more benign. Fourth, to promote toxicity, aggregated species often sequester large metastable proteins with unstructured regions, which typically occupy key nodes in functional networks that are crucial for cell viability (Olzscha et al., 2011). Potentiated Hsp104 variants could preferentially disaggregate and reactivate these loosely associated metastable proteins and thereby rescue toxicity without completely resolving the underlying aggregates that are formed by the human disease protein. These possibilities are not mutually exclusive, and indeed could even synergize to rescue toxicity.

We have also further elucidated the mechanism for the broad substrate specificity of potentiated Hsp104. Hsp104^WT^ recognizes unfolded substrates for disaggregation (DeSantis et al., 2012; Doyle et al., 2007). Potentiated Hsp104 maintains this mode of substrate recognition, and neither Hsp104^WT^ nor Hsp104^A503V^ unfold untagged, fully folded GFP. Both Hsp104^WT^ and Hsp104^A503V^ unfold RepA_1–70_-GFP, although Hsp104^A503V^ does so more rapidly and in the presence of ATP, whereas Hsp104^WT^ requires a mixture of ATP and ATPγS. However, for substrates with shorter unfolded tracts, Hsp104^WT^ and Hsp104^A503V^ respond differently. Hsp104^WT^ weakly unfolds 6-HIS-GFP, a substrate with a relatively short unstructured tract, whereas Hsp104^A503V^ robustly unfolds 6-HIS-GFP at the same rate that it unfolds RepA_1–70_-GFP. This enhanced substrate recognition could enable potentiated Hsp104 variants to disaggregate structures that would go unnoticed by Hsp104^WT^. This difference in substrate selection probably empowers potentiated Hsp104 to disaggregate recalcitrant substrates, as well as those that are only slightly destabilized or display only short unstructured tracts, whereas Hsp104^WT^ is unable to remodel these substrates.

Our results also help to explain several puzzling features of potentiated Hsp104. Although Hsp104^A503V^ confers tolerance to thermal stress (e.g. 50°C) at levels similar to Hsp104^WT^, it confers a growth defect at 37°C (Jackrel et al., 2014; Schirmer et al., 2004). We hypothesize that this might be due in part to the altered mode of substrate selection that we have observed for Hsp104^A503V^. Under the extreme conditions of thermal stress, many proteins in the yeast proteome misfold and aggregate (Cashikar et al., 2005). Here, Hsp104^WT^- or Hsp104^A503V^-mediated protein disaggregation and reactivation restores proteostasis (Jackrel et al., 2014; Parsell et al., 1994; Sanchez and Lindquist, 1990; Schirmer et al., 2004). However, under less stressful conditions (e.g. 37°C), many yeast proteins are likely to populate mildly destabilized or metastable states but are not entirely inactivated or misfolded, as they would be under conditions of more severe thermal stress. In this situation, we suggest that potentiated Hsp104^A503V^ inappropriately unfolds these metastable substrates. Excessive and inappropriate unfolding would overwhelm the proteostasis network and cause toxicity. This hypothesis is supported by the observation that Hsp104^A503V^ toxicity at 37°C is partially suppressed by overexpression of Hsp90 (Schirmer et al., 2004), a molecular chaperone that specializes in the recognition and stabilization of metastable folds that are likely to be the quarry of Hsp104^A503V^ (Taipale et al., 2010). Indeed, we suggest that the short unstructured tracts that are recognized by Hsp104^A503V^ might be similar to the clefts of the inherently metastable client proteins of Hsp90 (Pearl and Prodromou, 2006).

We have demonstrated that potentiated Hsp104 variants suppress the toxicity of a diverse array of substrates, including mutant variants that are implicated in disease. To further demonstrate the feasibility of applying Hsp104 to reversing disease in humans, it will be essential to develop substrate-optimized Hsp104 variants to avoid off-target effects. Certain potentiated variants that we have developed suppress toxicity more strongly than others. Many factors might contribute to these differences, such as tighter binding affinity or enhanced substrate translocation. Alternatively, certain Hsp104 variants could be tuned to recognize disordered versus amyloid aggregates. Variants that suppress the toxicity of certain substrates more than others could be the best candidates to develop further.

## MATERIALS AND METHODS

### Yeast strains, media and plasmids

All yeast strains were W303aΔ*hsp104* (*MATa*, *can1-100*, *his3-11*,*15*, *leu2-3*,*112*, *trp1-1*, *ura3-1*, *ade2-1*) (Sanchez and Lindquist, 1990). Yeast were grown in rich medium (YPD) or in synthetic medium lacking the appropriate amino acids. The medium was supplemented with 2% glucose, raffinose or galactose. Vectors encoding TDP-43, FUS, α-syn, EWSR1 (untagged and GFP-tagged) and TAF15 (pAG303GAL-TDP-43, pAG413GAL-FUS, pAG423GAL-α-syn-YFP, pAG423GAL-EWSR1, pAG426GAL-GFP-EWSR1 and pAG426GAL-TAF15, respectively) were kindly provided by Aaron Gitler and Martin Duennwald (Johnson et al., 2008; Johnson et al., 2009; Sun et al., 2011). pRS416GAL-Hsp104 variants have been described previously (Jackrel et al., 2014).

### Yeast transformation and spotting assays

Yeast were transformed according to standard protocols using polyethylene glycol and lithium acetate (Gietz and Schiestl, 2007). For the TDP-43 strains, yeast were transformed with linearized pAG303GAL-TDP-43^WT^ or its variants. Single colonies were selected, grown and screened for toxicity on galactose medium. Colonies showing a strong toxicity phenotype were selected and subsequently transformed with the pRS416GAL-Hsp104 plasmids. For the FUS and α-syn strains, yeast were co-transformed with the appropriate plasmids. Strains were then grown to saturation overnight in raffinose-supplemented dropout medium at 30°C. Cultures were serially diluted fivefold and spotted in duplicate onto synthetic dropout medium containing glucose or galactose. Plates were analyzed after growth for 2–3 days at 30°C.

### Immunoblotting

Yeast were grown and induced in galactose-containing medium for 5 h (TDP-43, FUS and EWSR1) or 8 h (α-syn). Cultures were normalized to A_600nm_=0.6, 6 ml of cells was then harvested and treated in 0.1 M NaOH for 5 min at room temperature, and cell pellets were resuspended into 100 μl 1×SDS sample buffer and boiled. Cleared lysates were separated using SDS-PAGE (4–20% gradient, Bio-Rad) and were then transferred to a PVDF membrane. Membranes were blocked using LI-COR blocking buffer for 1 h at room temperature. Primary antibody incubations were performed at 4°C overnight. Antibodies used were: anti-GFP monoclonal (Roche Applied Science), anti-TDP-43 polyclonal (Proteintech), anti-FUS polyclonal (Bethyl Laboratories), anti-EWSR1 monoclonal (Santa-Cruz Biotechnology), anti-Hsp104 polyclonal (Enzo Life Sciences) and anti-3-phosphoglycerate kinase (PGK) monoclonal (Invitrogen). Blots were processed using LI-COR Odyssey Fc Imaging system.

### Fluorescence microscopy

Yeast were induced in galactose-containing medium for 5 h (TDP-43, FUS and EWSR1) or 8 h (α-syn) at 30°C. Live cells were harvested and analyzed using fluorescence microscopy (FUS, EWSR1 and α-syn). For imaging TDP-43, cells were harvested and fixed in 70% ethanol for 10 min at room temperature. Cells were then washed in water and stained with 4′,6-diamidino-2-phenylindole in Vectashield mounting medium (Vector Laboratories) to visualize nuclei. For α-syn, cells were categorized as displaying membrane localization or containing cytoplasmic aggregates. Representative images are shown and quantification is the result of 3–4 independent experiments, each containing at least 150 cells per treatment. Images were collected at 100× magnification and processed using ImageJ software.

### Sedimentation assays

Sedimentation assays were performed as described previously (Jackrel et al., 2014). Briefly, yeast were grown and induced in galactose-containing medium for 8 h. Cultures were normalized to A_600nm_=0.6, and 100 ml of cells was harvested. The cell pellets were resuspended in 10 ml of yeast lysis buffer (30 mM HEPES-KOH pH 7.3, 150 mM NaCl, 1% glycerol, 0.5% Triton X-100, 5mM EDTA, 1mM DTT, 1mM PMSF) that was supplemented with yeast protease inhibitor cocktail (Sigma). Cells were disrupted using three passes through a French Press (Emulsiflex C-3) and cleared by centrifugation (6000 ***g*** for 5 min, 4°C). An aliquot of cleared lysate was reserved as total protein, and another aliquot was separated into a soluble and pellet fraction by centrifugation (100,000 ***g*** for 15 min, 4°C). Fractions were then resolved using SDS-PAGE and processed for quantitative immunoblot.

### Protein purification

Potentiated Hsp104 variants were purified as described previously (Jackrel et al., 2014). Hsp104 concentrations refer to the hexamer concentration. GroEL^trap^ was purified as described previously (Doyle et al., 2007). RepA_1–70_-GFP was purified by expressing N-terminally HIS-tagged protein in *Escherichia coli* followed by recovery from inclusion bodies in 6 M urea. Urea was removed by dialysis, and the protein was applied to Ni-NTA beads. The eluted protein was used with the tag. 6-HIS-TEV-GFP was purified by expressing the protein in *E. coli* and purification using standard techniques. The protein was also cleaved using TEV-protease, and the tag was removed by purifying the protein over Ni-NTA beads. FUS, TAF15 and EWSR1 were purified as described previously (Couthouis et al., 2012; Couthouis et al., 2011; Sun et al., 2011). Sse1, Ssa1 and Ydj1 were purified as described previously (Raviol et al., 2006; Shorter and Lindquist, 2008).

### Protein disaggregation

To generate pure FUS, TAF15 and EWSR1 aggregates, GST-TEV-FUS, GST-TEV-TAF15 or GST-TEV-EWSR1 (6 μM) were incubated with TEV protease (Life Sciences) in assembly buffer (50 mM Tris-HCl pH 7.4, 50 mM KCl, 5 mM MgCl_2_, 0.2 M trehalose and 20 mM glutathione) for 90 min at 25°C without agitation, by which time all the FUS, TAF15 and EWSR1 had been converted to the aggregated state (Couthouis et al., 2012; Couthouis et al., 2011; Sun et al., 2011). FUS, TAF15 or EWSR1 aggregates (3 μM monomer) were then incubated for 60 min at 30°C with the indicated combination of Hsp104^WT^ or Hsp104^A503S^ (1 μM), with or without Ssa1 (1 μM), Ydj1 (0.44 μM) and Sse1 (0.26 μM), plus ATP (10 mM) and the regeneration system components (20 mM creatine phosphate and 0.5 mM creatine kinase). Disaggregation was assessed using turbidity (absorbance at 395 nm) (Couthouis et al., 2012; Couthouis et al., 2011; Sun et al., 2011).

### RepA_1–70_-GFP unfolding

RepA_1–70_-GFP unfolding was performed as described previously (Doyle et al., 2007), with modifications. Briefly, 30 μl reactions were prepared on ice in buffer containing 20 mM HEPES-KOH pH 7.4, 250 mM KCl, 10 mM MgCl_2_, 5% glycerol, 0.5 mM DTT, 20 μg/ml BSA, 0.005% (vol/vol) Triton X-100, 1 mM creatine phosphate, 0.25 μM creatine kinase and 10 mM nucleotide as indicated (10 mM ATP for Hsp104^A503V^, 7.5 mM ATP plus 2.5 mM ATPγS for Hsp104^WT^), 2.1 μM Hsp104 hexamer and 1.5 μM GroEL^trap^. Reactions were transferred to 384-well plates and initiated by the addition of 0.7 μM RepA_1–70_-GFP, 6-HIS-GFP or GFP, and fluorescence was monitored using a Tecan Safire^2^ microplate reader.
